# Predicting the Distribution Pattern of Small Carnivores in Response to Environmental Factors in the Western Ghats

**DOI:** 10.1371/journal.pone.0079295

**Published:** 2013-11-14

**Authors:** Riddhika Kalle, Tharmalingam Ramesh, Qamar Qureshi, Kalyanasundaram Sankar

**Affiliations:** 1 Wildlife Institute of India, Dehra Dun, Uttarakhand, India; 2 Department of Landscape Ecology, Wildlife Institute of India, Dehra Dun, Uttarakhand, India; 3 Department of Habitat Ecology, Wildlife Institute of India, Dehra Dun, Uttarakhand, India; Estacion Experimental de Zonas Áridas (CSIC), Spain

## Abstract

Due to their secretive habits, predicting the pattern of spatial distribution of small carnivores has been typically challenging, yet for conservation management it is essential to understand the association between this group of animals and environmental factors. We applied maximum entropy modeling (MaxEnt) to build distribution models and identify environmental predictors including bioclimatic variables, forest and land cover type, topography, vegetation index and anthropogenic variables for six small carnivore species in Mudumalai Tiger Reserve. Species occurrence records were collated from camera-traps and vehicle transects during the years 2010 and 2011. We used the average training gain from forty model runs for each species to select the best set of predictors. The area under the curve (AUC) of the receiver operating characteristic plot (ROC) ranged from 0.81 to 0.93 for the training data and 0.72 to 0.87 for the test data. In habitat models for *F. chaus*, *P. hermaphroditus*, and *H. smithii* “distance to village” and precipitation of the warmest quarter emerged as some of the most important variables. “Distance to village” and aspect were important for *V. indica* while “distance to village” and precipitation of the coldest quarter were significant for *H. vitticollis*. “Distance to village”, precipitation of the warmest quarter and land cover were influential variables in the distribution of *H. edwardsii*. The map of predicted probabilities of occurrence showed potentially suitable habitats accounting for 46 km^2^ of the reserve for *F. chaus*, 62 km^2^ for *V. indica*, 30 km^2^ for *P. hermaphroditus*, 63 km^2^ for *H. vitticollis*, 45 km^2^ for *H. smithii* and 28 km^2^ for *H. edwardsii*. Habitat heterogeneity driven by the east-west climatic gradient was correlated with the spatial distribution of small carnivores. This study exemplifies the usefulness of modeling small carnivore distribution to prioritize and direct conservation planning for habitat specialists in southern India.

## Introduction

Predictive habitat modeling and mapping are useful in conservation planning, detecting distributional changes from monitoring data and quantifying variation in species performance towards several controlling factors. Predictive distribution models for small carnivores only exist at large geographical scales [1]–[3]. However, studies performed at smaller scales are essential for accurate understanding of ecological interactions and identifying drivers of species distributions for immediate conservation actions [4]. The Western Ghats has been listed as a World Heritage Site by the United Nations Educational, Scientific and Cultural Organisation (UNESCO). It is one of the world’s eight “hottest hotspots” of biodiversity [5] including globally significant populations of 13 small carnivore species; *viz* four species of small felids, four herpestids, four viverrids and five mustelids. Field investigations are often challenging particularly in dense tropical habitats when carnivores are small-sized, elusive, secretive, arboreal, and purely nocturnal therefore displaying low detection probabilities. Scientific information on small carnivores in India is still deficient in most parts of the country [1], [6]–[8]. Smaller carnivores lack the glamour and attention that conservationists and managers seek in mega-carnivores as conservation tools and appropriate flagship species. Conducting focal studies on small carnivores is a daunting task as procuring government funds for conservation measures represents a huge challenge.

As specialization and resource selectivity is generally stronger in small carnivores than large carnivores [8], they may serve as useful indicator species in the preservation of keystone habitats. Knowledge on spatial scale and landscape heterogeneity is integral to the understanding of species habitat associations, though at present, much data on their relationships is drawn from a few long-term [9], [10] and short-term studies [11]–[13], anecdotes [14], sightings [15], observations [16], rapid surveys [17], [18] and phylogenetic information [1]. Anthropogenic activities such as urbanization, commercial plantations, and intensive agricultural practices have led to severe habitat loss and fragmentation of tropical forests in southern India. Major threats to small wild cats include habitat destruction, fragmentation, poaching, and hybridization with domestic cats [19]. Mongooses are often hunted for meat by several tribes and local villagers and they are even trapped for fur used in shaving brushes, paint brushes, and good luck charms [20]. Viverrids are severely threatened due to hunting by indigenous local tribal communities. They are often trapped for meat; animal parts are used for making local medicines, aphrodisiacs, and in traditional rituals [21].

In the biogeographic zones of India, small carnivores (habitat specialists or generalists) are sympatric depending upon their habitat utilization and niche characteristics. Due to data limitations, most of the distribution maps on small carnivores in Indian literature were created traditionally by compiling locality records in combination with general knowledge and expert opinion on potential habitat. A more rigorous and widely used approach of species distribution modeling is necessary to get a quantitative perspective of complex factors controlling animal distribution, including an assessment of uncertainty in modeling rare and cryptic species. Alternative procedures especially those combining sight-resight (capture-recapture) and occupancy modeling by incorporating covariates may be just as effective in describing the geographic range and habitat characteristics of the target species [22], [23]. Although presence/absence models are frequently used to predict species distributions, there is a common problem related to uncertainty in determining absences [24]. In situations particularly dealing with rare and cryptic species that are arduous to survey, ecologists can either model presence/pseudo-absence (or background) data [25], or model presence-only data, e.g.: [26]. Models based on randomly generated pseudo-absences are expected to have lower predictive power than those built with actual absences [27]. For modeling rare species without true absence data, pseudo-absences may be particularly appropriate [28]. A presence-only approach may still be favorable since the need for truly comprehensive and exclusive absence, which although is a requirement, is usually not met by most biodiversity data. The maximum entropy (MaxEnt) software does not require direct absence data. It produces a good prediction of species distribution [29] and models appear to be robust even when few occurrence records or incomplete data are available [30], [31].

Habitat suitability maps are widely applied in conservation biology and wildlife management to facilitate protection and restoration of critical habitat [32]. It is imperative to investigate how small carnivores in southern tropical forests of India respond or relate to variables within a Protected Area setting that generally hold acres of continuous optimal habitat. With this view, the present study aimed at utilizing MaxEnt software [32] to model the distribution and habitat suitability of six species of small carnivores: the jungle cat *Felis chaus* (Schreber, 1777), small Indian civet *Viverricula indica* (É. Geoffroy Saint-Hilaire, 1803), common palm civet *Paradoxurus hermaphroditus* (Pallas, 1777), stripe-necked mongoose *Herpestes vitticollis* (Bennett, 1835), ruddy mongoose *Herpestes smithii* (Gray, 1837), and grey mongoose *Herpestes edwardsii* (É. Geoffroy Saint-Hilaire, 1818) in Mudumalai Tiger Reserve (Mudumalai), Western Ghats, India. The goals of the study were to generate habitat suitability models to 1) predict small carnivore distribution using environmental variables and 2) identify key environmental variables associated with species occupancy and suitable habitats where species are likely to be found; to guide managers for local conservation strategies.

## Materials and Methods

### Ethics Statement

All permissions to carry out field research were obtained from the Office of the Chief Wildlife Warden, Tamil Nadu state under the provisions of the Wildlife (Protection) Act, 1972, and the Guidelines for Scientific Research in Protected Areas, Ministry of Environment and Forests, Government of India. Wherever observational investigations were made during vehicle transects, no animals were harmed.

### Study Site

**Figure 1:**
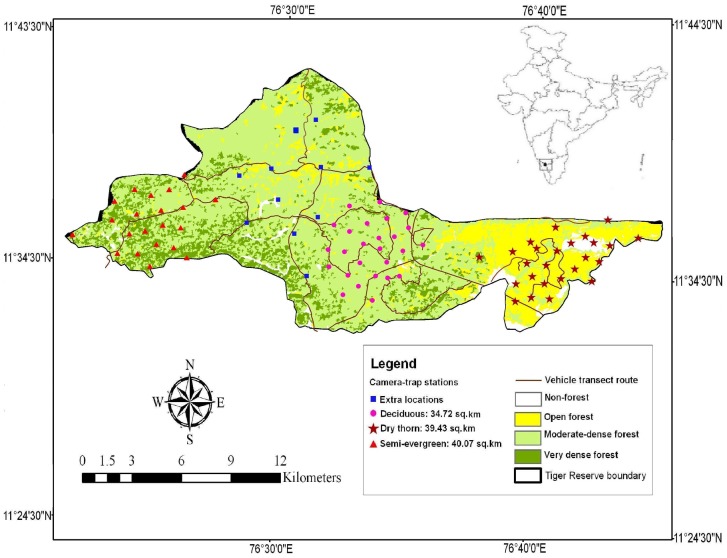
Location of the study area showing the spatial distribution of camera-traps and vehicle transect routes in Mudumalai Tiger Reserve (2010 and 2011).

The study was carried out in Mudumalai Tiger Reserve (11° 32′–11° 43′ N; 76° 22′–76°45′ E), located in the centre of the Nilgiri Biosphere Reserve at the tri-junction of Tamil Nadu, Karnataka, and Kerala states in India (Figure 1). The topography is undulating with elevation ranging from 960 to 1,266 m. The spatio-temporal rainfall gradient from east to west brings corresponding changes in the vegetation. The 321 km^2^ reserve comprises diverse forest types such as dry thorn, dry deciduous, moist deciduous, semi-evergreen, moist bamboo brakes and riparian fringe forests [33]. The climate is monsoonal, with one dry season (January to April) and two wet seasons (May to August and September to December) seasons. Eastern areas face the shortest periods of the heaviest rains (1,000–2,000 mm). Mean temperature ranged from 15°C to 32°C in the dry season, 17°C to 30°C in the first wet season and 16°C to 26°C in the second wet season (Centre for Ecological Sciences, Indian Institute of Science). Primary threats to the region arise from severe human pressures (cattle grazing, cultivations, settlements, collection of fuel wood and non-timber forest products, etc.), due to the ever expanding human population.

### Field Data Collection

**Figure 2:**
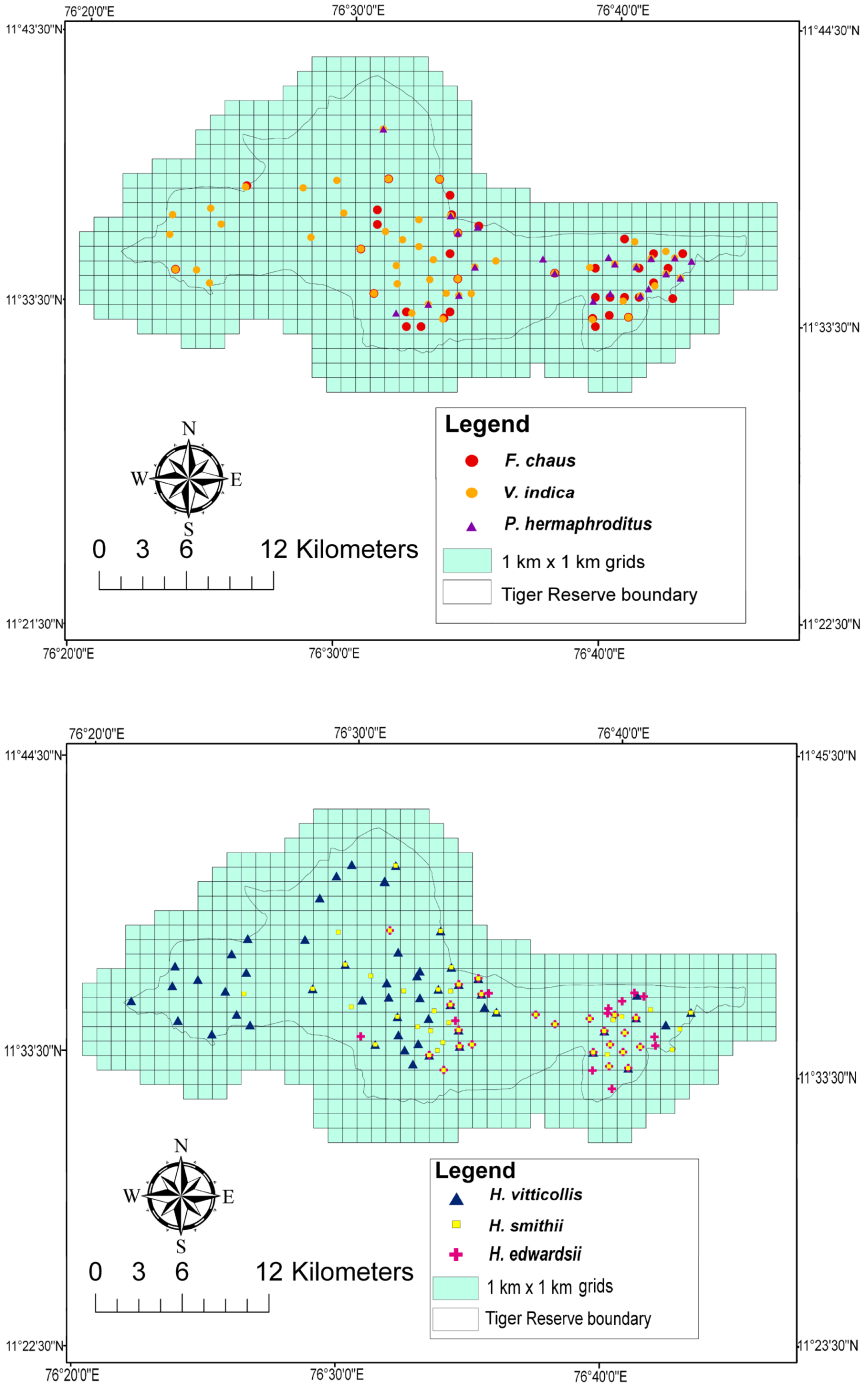
Spatially unique localities of six small carnivore species in Mudumalai Tiger Reserve (2010 and 2011).

We compiled presence-only records of six study species from field surveys by camera-trapping and vehicle transects in Mudumalai (2010 and 2011). The reserve was overlaid with 674 grids of 1 km^2^ each, including 2 km of buffer area outside Mudumalai using the Geographic Information System (GIS) in ArcGIS 9.3 (Environmental Systems Research Institute (ESRI), Inc., Redlands, CA, USA)). Grid size was defined on the basis of home range of small carnivore species; 0.1–1.5 km^2^ for *H. edwardsii*, 0.65 km^2^ for *V. indica* [13], and 14.1 ha for *P. hermaphroditus* [11]. Although no home range estimates are available for small cats in India, they are known to have larger home ranges than herpestids and viverrids. We selected 114 km^2^ within the intensive study area including three sampling zones in deciduous (35 km^2^), semi-evergreen (40 km^2^) and dry thorn forests (39 km^2^). Camera trap survey was conducted for two years in the intensive study area. The deciduous and dry thorn forests were sampled in the dry and wet seasons while the semi-evergreen forest was sampled only in the dry season due to inaccessibility and logistic constraints in the wet season. We selected the most suitable sites likely to trap all species of terrestrial small carnivores based on preliminary sign surveys of their tracks, scats, carcasses, interviews with local people and park guards. The choice of a camera trap location was thus decided after taking into account several ecological factors associated with species biology from previous research [1], [6], [9], [13], [16], [18], [43], [45]. We deployed passive-infrared camera traps in a systematic grid 1×1 km^2^ using DEERCAM DC300 (DeerCam, Park Falls, USA) and STEALTHCAM (Bedford, Texas, USA). The mean inter-camera trap distance was 1.31 km. Each year we setup 26 camera trapping stations in the deciduous forest, 21 in the semi-evergreen forest and 25 in the dry thorn forest. We also setup 11 camera-trap stations randomly, at sites outside the intensive area to maximize our data set and increase the sampling area (Figure 1). Stations consisted of two independently operating passive-infrared cameras mounted on opposite sides of a trail or dirt road to get photocaptures of small carnivores. Cameras were approximately 25 cm above the ground and set to be active for 24 h/day. No bait or lure was used at any location to attract animals. The photocapture delay was set to 1 min and sensitivity was set to high. Stations were sampled for 30 days during which they were checked on an average of every three days to ensure continued operation. Batteries and film were replaced when necessary. Vehicle transect routes were well distributed within the reserve allowing us to survey *c*.107 km^2^. Transects ranging from 15 to 23 km were each surveyed bimonthly during the study period in the early morning and late evening at a speed of 20 km/hr. All species sightings were pooled across surveys for each transect. All species records collated from both field techniques were pooled across years, mapped in ArcGIS 9.3 (ESRI), and overlaid with 1 km^2^ grid cells. We considered all overlapping records as a single record, hence only spatially independent locations were selected for further analysis resulting in a final number of 36 point localities for *F. chaus*, 51 for *V. indica*, 22 for *P. hermaphroditus*, 55 for *H. vitticollis*, 51 for *H. smithii* and 35 for *H. edwardsii* (Figure 2).

### Environmental Predictors

Ecological requirements of small carnivores provide substantial evidence that their distribution is determined by resources at the home-range scale. Predictors included bioclimatic variables, forest and land cover types, topography, vegetation indices and anthropogenic variables (Table 1). We initially considered 19 ‘bioclimatic’ variables from WORLDCLIM database (www.worldclim.com) [34], and elevation layer at 1 km^2^-resolution from Shuttle Radar Topography Mission (SRTM) elevation database (http://srtm.csi.cgiar.org). Mean slope and aspect was calculated from elevation layer using Surface analysis tool from Spatial Analyst toolbox in ArcGIS 9.3 (ESRI). The slope layer had values measured from 0° to 90° for each 1 km^2^ pixel. Aspect was transformed to represent incident radiation [28], [35], [36]. The transformation uses the compass value given by the elevation layer (0–360°), normalizes it between 0 and 1 and then takes the absolute value, which serves to fold the aspect, giving equal value to aspects that are equidistant east or west of the meridian. This transformed value is called “directionality” since it is ∼0 when aspect is towards the north and ∼1 when near south. The calculation is given by:
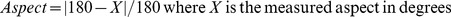



**Table 1:** Predictor variables tested for habitat suitability modeling of small carnivores in Mudumalai Tiger Reserve.

Variable		Code	Source	Type
**Climate**	Bio1 = Annual Mean Temperature (°C)		Worldclim	Continuous
	Bio 2 = Mean Diurnal Range (Mean of monthly (max temp – min temp) (°C)	Bio2		
	Bio 3 = Isothermality (Bio2/Bio7)	Bio3		
	Bio 4 = Temperature Seasonality (standard deviation x100) (°C)			
	Bio 5 = Max Temperature of Warmest Month (°C)	Bio5		
	Bio 6 = Min Temperature of Coldest Month (°C)			
	Bio7 = Temperature Annual Range (Bio5–Bio6) (°C)	Bio7		
	Bio 8 = Mean Temperature of Wettest Quarter (°C)			
	Bio 9 = Mean Temperature of Driest Quarter (°C)			
	Bio 10 = Mean Temperature of Warmest Quarter (°C)			
	Bio 11 = Mean Temperature of Coldest Quarter (°C)			
	Bio 12 = Annual Precipitation (mm)			
	Bio 13 = Precipitation of Wettest Month (mm)			
	Bio 14 = Precipitation of Driest Month (mm)			
	Bio 15 = Precipitation Seasonality (Coefficient of Variation) (Fraction)			
	Bio 16 = Precipitation of Wettest Quarter (mm)			
	Bio 17 = Precipitation of Driest Quarter (mm)			
	Bio 18 = Precipitation of Warmest Quarter (mm)	Bio18		
	Bio 19 = Precipitation of Coldest Quarter (mm)	Bio19		
**Elevation (m)**			SRTM	Continuous
**Slope (**°**) and Aspect (**°**)**			Calculated from SRTM Digital Elevation Model	Continuous
**Forest cover type**	1 = water bodies		Forest Survey of India	Categorical
	2 = non-forest			
	3 = scrub			
	4 = open forest			
	5 = dense forest			
	6 = very dense forest			
**Land-cover categories**	1 = tropical evergreen			Categorical
	2 = subtropical evergreen			
	8 = moist deciduous			
	9 = dry deciduous			
	16 = degraded forest			
**Distance to the nearest village/tribal settlement (m)**		D2V	field data(GPS locations)	Continuous
**Topography Wetness Index**		WI	USGS	Continuous
**Actual evapo-transpiration**		AET	CGIAR-CSI	Continuous
**Distance to the nearest water source (m)**		D2W	DIVAGIS	Continuous
**Normalized Difference Vegetation Index**		NDVI	AVHRR	Continuous

Forest cover map was derived from Forest Survey of India and categorized into 6 classes. The land use land-cover map at 1∶250000 scale was derived from DIVAGIS (version 7.1.7.2, http://www.diva-gis.org) where original data was resampled to a 30 seconds grid (source-GLC2000) and further classified into 5 categorical variables. Actual evapo-transpiration (AET) is the effective quantity of water that is removed from the soil due to evaporation and transpiration processes. We derived mean AET values (mm) (http://www.cgiar-csi.org) [37], at 30 arc-seconds (∼1 km^2^). Surface water bodies (rivers and streams) were extracted from DIVAGIS (version 7.1.7.2, http://www.diva-gis.org). The Euclidean-distance tool was used to create a raster “distance to” (km) layer for the closest water source and village/tribal settlement (with 1 km buffer) such that each pixel was assigned a value of distance to water and village/tribal settlement. Monthly Normalized Difference Vegetation Index (NDVI) was downloaded from Advanced Very High Resolution Radiometer (AVHRR) sensor where a value of zero means no green vegetation and close to +1 (0.8–0.9) indicates the highest possible density of green leaves. All environmental variables were re-sampled at a resolution of ∼1 km^2^ using the Zonal Statistic tool in the Spatial Analyst toolbox in ArcGIS 9.3 (ESRI).

All environmental layers were converted to GRID (raster) format and resampled to 1 km resolution using the raster calculator tool in the ArcGIS Spatial Analyst extension. Using many correlated variables may result in over-parameterization and reduce the predictive power and interpretability [38]. Multicollinearity was checked for all combinations of environmental variables. Since elevation, bioclimatic variables and NDVI were highly correlated (R^2^ = ≥0.7), we retained only those variables which showed little correlation with other predictors; bio3 = isothermality (mean diurnal temperature range/[maximum temperature of the warmest month/minimum temperature of the coldest month] in °C), bio18 = precipitation of the warmest quarter (mm), bio19 = precipitation of the coldest quarter (mm), mean NDVI (March), mean NDVI (June) and mean NDVI (July). These variables were selected due to their probable ecological significance (Table S1). All retained datasets were then exported as ASCII files in MaxEnt software, version 3.3.3 k (http://www.cs.princeton.edu/~schapire/maxent).

### Species Distribution Modeling and Validation

MaxEnt is a machine learning algorithm that estimates the most uniform distribution (maximum entropy) across the study area given the constraint that the expected value of each environmental variable under this estimated distribution matches its empirical average [32] and frequently performs better than other presence-only modeling techniques [29]. The modeled probability is a ‘Gibbs’ distribution (i.e. exponential in a weighted sum of the features) and the model logistic outputs have a natural probabilistic interpretation representing degrees of habitat suitability (0 = unsuitable to 0.99 = best habitat). Like most maximum-likelihood estimation approaches, the MaxEnt algorithm *a priori* assumes a uniform distribution and performs a number of iterations in which the weights associated with environmental variables, or functions thereof, are adjusted to maximize the average probability of the point localities expressed as the training gain. These weights are then used to compute MaxEnt distribution over the entire geographic space. Consequently, this distribution expresses the suitability of each grid cell as a function of the environmental variables for that grid cell.

A set of ASCII environmental layers and a csv file of presence locations of species were used to produce probability maps that predict the potential distribution of a species. The measure of fit implemented by MaxEnt is the area under the curve (AUC) of a receiver operating characteristic (ROC) plot (ranging from 0.5 = random to 1 = perfect discrimination). The final reduced data set converged to a total of 14 environmental layers were projected to the UTM zone to match their coordinates, clipped to the extent of the boundary along with 2 km buffer, and entered with species occurrence data into MaxEnt version 3.3.3 (http://www.cs.princeton.edu/~schapire/maxent). For all models run in this study, we used the MaxEnt default settings for regularization and selecting the feature classes (functions of environmental variables). These include linear, quadratic, product, threshold and hinge features, depending on the number of point localities. Respectively, they constrain means, variances, and covariances of respective variables to match their empirical values [32]. It should be noted that the model algorithm (MaxEnt) used in this study is largely robust to covariance among variables, and that data reduction was performed mainly to improve interpretation [39]. The program was set to run 1,000 iterations with a convergence threshold of 0.00001, a regularization multiplier of 1, a maximum of 10,000 background points, the output grid format as “ logistic,” algorithm parameters set to “auto features,” and all other parameters at their default settings [40]. We had the program randomly withhold 20%, 30%, 40% and 50% of the presence locations to test the performance of each model. The split-sample procedure was repeated ten times with the aforementioned settings and thus 40 models were calibrated for each species. Inference was based on average estimates of AUC, predictor importance, and prediction maps (mean probability of occurrence) calculated from these models.

### Variable Contribution and Response Curves

We considered MaxEnt’s heuristic estimates of the relative contribution of environmental variables to the models and the results of jackknife analysis for each environmental layer [40]. There are two methods to assess the contributions of environmental factors to models: 1) percentage contribution and permutation importance and 2) the jackknife test. These percent contribution values are only heuristically defined: they depend on the particular path that the MaxEnt code uses to get to the optimal solution, and a different algorithm could get to the same solution via a different path, resulting in different percent contribution values. In addition, when there are highly correlated environmental variables, the percent contributions should be interpreted with caution. The permutation importance measure depends only on the final MaxEnt model, not the path used to obtain it. The contribution for each variable is determined by randomly permuting the values of that variable among the training points (both presence and background) and measuring the resulting decrease in training AUC. A large decrease indicates that the model depends heavily on that variable. Values are normalized to give percentages.

To get alternate estimates of variable importance, we also ran a jackknife test. In this test, a number of models were created. Each variable was excluded in turn, and a model created with the remaining variables. Then a model was created using each variable in isolation. In addition, a model was created using all variables. For the variables with highest predictive value, response curves show how each of these environmental variables affects MaxEnt predictions [40]. The curves illustrate how the logistic prediction changes as each environmental variable is varied, while keeping all other environmental variables at their average sample value. The curves thus represent the marginal effect of changing exactly one variable. Each of the models was then re-run a second time, after selecting only those variables that contributed at least 2% to the initial model result. This methodology reduced the total number of variables used in the analysis.

## Results

### Significant Explanatory Variables and Model Performance

For all models, the area under the curve (AUC) of the receiver operating characteristic plot (ROC) was high for the training data (ranging from 0.81–0.93) and test data (ranging from 0.72–0.87, Table 2). AUC values in this range are considered informative [40] and indicative of good accuracy [41]. In *F. chaus* models based on percent contribution, “distance to village” was the most important variable followed by precipitation of the warmest quarter (bio18) (30.4% and 23.73%, respectively). Based on permutation importance, bio18 was the most significant variable (41.6%) followed by “distance to village” (32.26%) in *F. chaus* models (Figure S1(A)). In *V. indica* models based on percent contribution and permutation importance, “distance to village” had the greatest influence (65.04% and 52.18%, respectively) followed by aspect (11.12% and 16.84%, respectively (Figure S1(B)). In *P. hermaphroditus* models based upon percent contribution and permutation importance, bio18 was the most influential variable (55.55% and 61.71%, respectively) followed by “distance to village” (21.34% and 32.61%, respectively) (Figure S1(C)). In *H. vitticollis* models, “distance to village” was the major determining factor for percentage contribution in projecting species range (54.91%), followed by forest cover (11.53%) and land cover (11.42%). For *H. vitticollis* “distance to village” was the most significant variable (47.39%) followed by precipitation of the coldest quarter (bio19) (20.38%) when permutation importance was considered (Figure S1(D)). In *H. smithii* models based on percent contribution, “distance to village” showed the greatest impact on species distribution (34.49%) followed by bio18 (29.4%) and models based on permutation importance showed that bio18 was the most influential variable (31.63%) followed by “distance to village” (31.63%, Figure S1(E)). In *H. edwardsii* models based on percent contribution, “distance to village” had the greatest influence in species distribution (31.45%) followed by bio18 (29.63%) and land cover (28.06%). At the same time, models based on permutation importance showed that bio18 showed the greatest impact (52.51%) followed by “distance to village” (31.1%, Figure S1(F)).

**Table 2:** Average estimates of Maxent distribution models for small carnivores in Mudumalai Tiger Reserve (2010 and 2011).

Species	Randomtest (%)*	Number of Training samples	Mean Regularized training gain	Mean Unregularized training gain	Mean Training AUC	Number of Test samples	MeanTest gain	MeanTest AUC	Mean AUC Standard Deviation
*F. chaus*	20	28	0.75	0.92	0.85	7	0.56	0.79	0.068
	30	25	0.75	0.96	0.86	10	0.55	0.80	0.061
	40	21	0.75	0.99	0.86	14	0.50	0.79	0.049
	50	18	0.79	1.04	0.86	17	0.39	0.78	0.048
	**Average**		**0.76**	**0.98**	**0.86**		**0.50**	**0.79**	**0.056**
*V. indica*	20	40	0.55	0.70	0.82	9	0.45	0.78	0.059
	30	35	0.60	0.78	0.83	14	0.24	0.73	0.054
	40	30	0.59	0.77	0.83	19	0.31	0.74	0.049
	50	25	0.62	0.84	0.84	24	0.24	0.73	0.043
	**Average**		**0.59**	**0.77**	**0.83**		**0.31**	**0.75**	**0.051**
*P. hermaphroditus*	20	18	1.04	1.30	0.89	4	0.77	0.85	0.050
	30	16	1.01	1.31	0.89	6	0.81	0.84	0.053
	40	14	0.91	1.19	0.88	8	0.79	0.85	0.040
	50	11	0.94	1.24	0.89	11	0.56	0.83	0.043
	**Average**		**0.97**	**1.26**	**0.89**		**0.73**	**0.84**	**0.046**
*H. vitticollis*	20	42	0.51	0.64	0.80	10	0.26	0.71	0.063
	30	37	0.49	0.64	0.80	15	0.38	0.74	0.053
	40	32	0.49	0.64	0.81	20	0.32	0.72	0.046
	50	26	0.52	0.72	0.83	26	0.24	0.71	0.042
	**Average**		**0.50**	**0.66**	**0.81**		**0.30**	**0.72**	**0.051**
*H. smithii*	20	38	0.84	1.03	0.87	9	0.74	0.83	0.062
	30	33	0.85	1.05	0.87	14	0.73	0.83	0.048
	40	29	0.84	1.07	0.88	18	0.70	0.82	0.042
	50	24	0.90	1.16	0.89	23	0.52	0.80	0.042
	**Average**		**0.86**	**1.08**	**0.88**		**0.67**	**0.82**	**0.048**
*H. edwardsii*	20	26	1.26	1.53	0.92	6	0.95	0.86	0.058
	30	23	1.24	1.54	0.93	9	0.93	0.87	0.043
	40	20	1.18	1.51	0.93	12	1.07	0.88	0.034
	50	16	1.28	1.63	0.93	16	0.88	0.86	0.036
	**Average**		**1.24**	**1.55**	**0.93**		**0.96**	**0.87**	**0.043**

The model performance was computed on different test data set.

The jackknife test of variable importance in *F. chaus*, *H. smithii* and *H. edwardsii* suitability models showed the highest gain when bio18 was used in isolation containing the most information when used alone, while “distance to village” decreased the gain the most when it was omitted, and therefore contained information not present in any other variable (Figure S1(A,E and F)). The jackknife test of variable importance in *V. indica* and *H. vitticollis* showed the greatest change when “distance to village” was used in isolation, indicating it contains the most useful and unique information in determining these species distributions (Figure S1(B and D)). In *P. hermaphroditus* the jackknife test indicated that bio18 was the most influential variable (Figure S1(C)).

### Response of Carnivores to Environmental Variables

**Figure 3:**
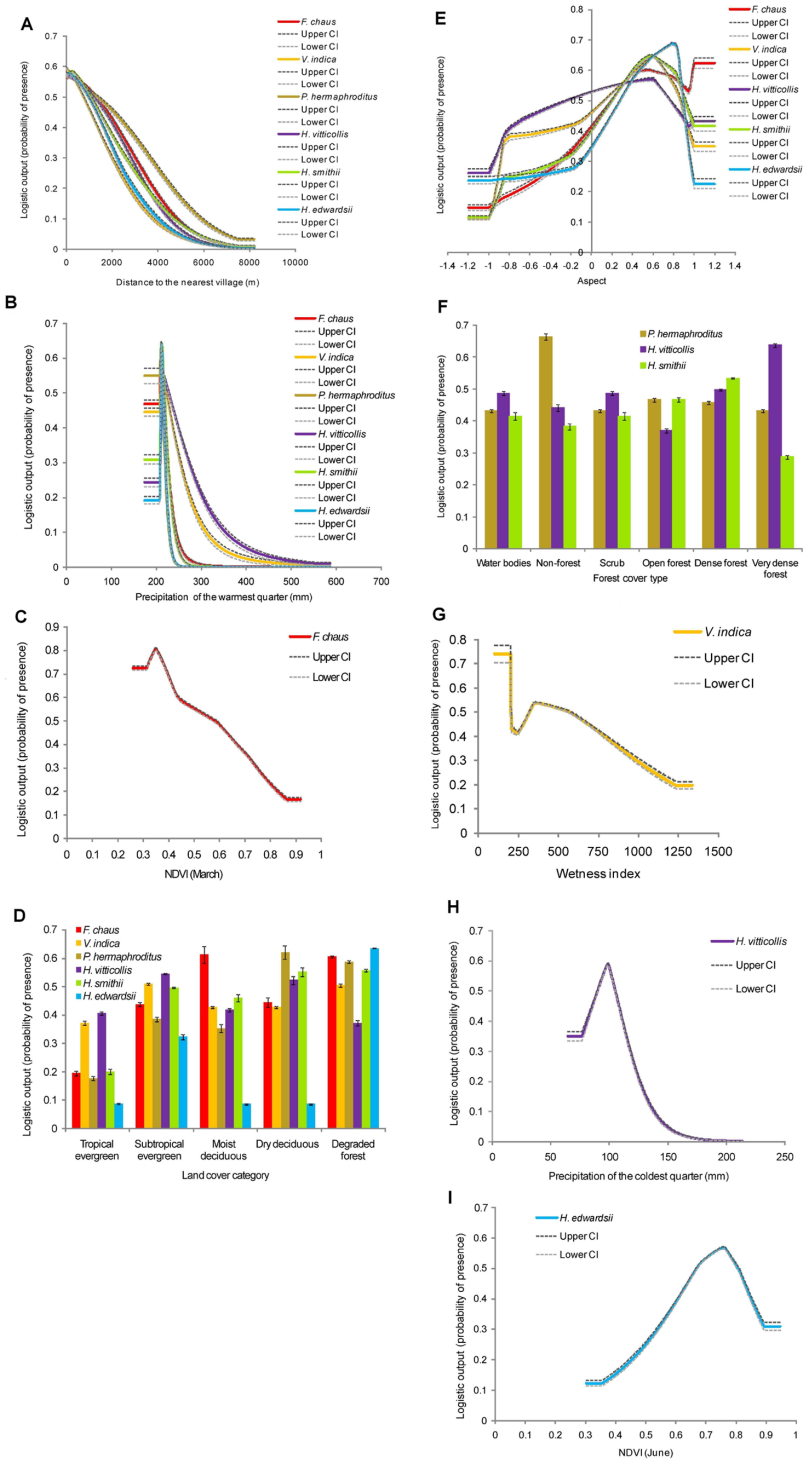
Response curves for the most significant predictors of habitat suitability of small carnivores according to the MaxEnt model. The response curve is shown in different colours. Each colour represents a different species. The dark grey and light grey dotted lines represent 95% confidence intervals from 40 replicated runs.

The response curve for “distance to village” showed a negative relationship with the logistic output (and thus habitat suitability) for all small carnivore species (Figure 3A). High probabilities of occurrence were skewed sharply towards low values of bio18 for *F. chaus* (at 213.4 mm), *P. hermaphroditus* (at 212.57 mm), *H. smithii* (at 214.23 mm), and *H. edwardsii* (at 213.4 mm, Figure 3B). Probabilities of *V. indica* and *H. vitticollis* were greatest towards low values of bio18 (at 212.57 mm and 215.88 mm, respectively) and gradually decreased with increasing values of bio18 (Figure 3B). NDVI (March) was negatively related to predicted presence of *F. chaus* (Figure 3C). Of the land cover categories, moist deciduous and degraded forests were highly suitable habitats for *F. chaus* presence (Figure 3D). Response curves for *F. chaus*, *V. indica*, *H. vitticollis*, *H. smithii* and *H. edwardsii* showed positive relationships with directionality (Figure 3E).

Sub-tropical evergreen and degraded forests were predicted as potentially suitable habitats for *V. indica* (Figure 3D). High probabilities of presence were predicted at low values of wetness index (WI) (103), slightly increasing at 246 and then gradually decreasing with increasing values of WI (Figure 3G). For *P. hermaphroditus*, highly suitable areas were projected in dry deciduous forest, degraded forest (Figure 3D) and non-forests (Figure 3F). *H. vitticollis* preferred sub-tropical evergreen forest, dry deciduous forests (Figure 3D), and very dense forest cover (Figure 3F). Its distribution was strongly constrained by bio19 (at 99.12 mm) (Figure 3H). *H. smithii* preferred dry deciduous, and degraded forests (Figure 3D), however, predicted suitability did not vary among forest cover (Figure 3F). *H. edwardsii* preferred degraded forests (Figure 3D) and areas with high canopy cover (NDVI (June) close to 0.76) (Figure 3I).

### Predicted Habitat Suitability Maps

**Figure 4:**
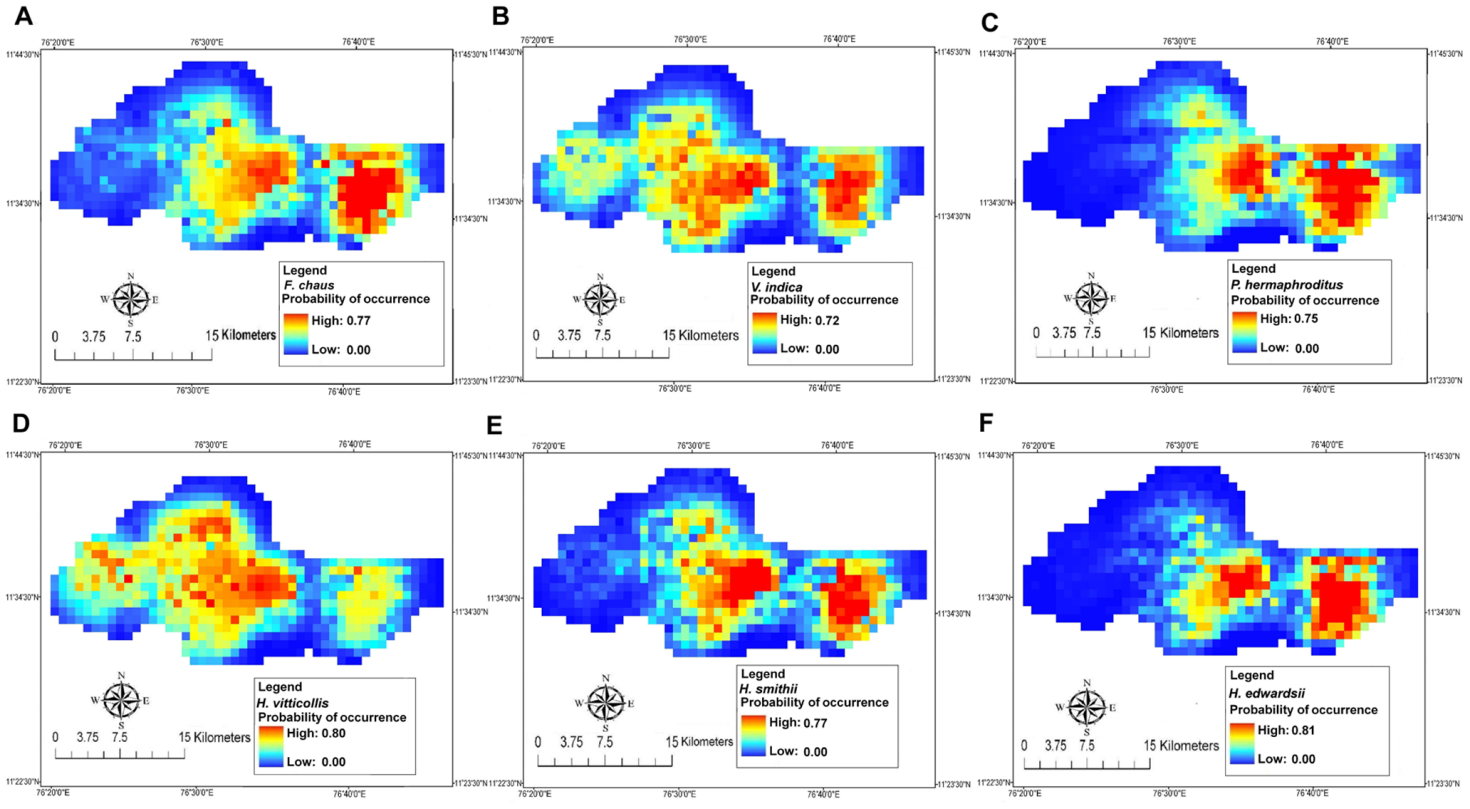
Habitat suitability maps of small carnivores based on MaxEnt models using environmental variables. Average MaxEnt predictions from 40 runs for each species at the scale of 1×1 km resolution. The predicted probability of presence, with values ranging from 0 to 1, is depicted by different colours. Using the MaxEnt logistic output, red colours indicate a higher “probability of occurrence” (suitability) while the blue colours indicate lower probabilities.

The MaxEnt model generated a map of predicted probabilities of occurrence showing potentially suitable habitats (≥0.6 probability of presence) accounting for 46 km^2^ of the reserve for *F. chaus* (Figure 4A), 62 km^2^ for *V. indica* (Figure 4B), 30 km^2^ for *P. hermaphroditus* (Figure 4C), 63 km^2^ for *H. vitticollis* (Figure 4D), 45 km^2^ for *H. smithii* (Figure 4E) and 28 km^2^ for *H. edwardsii* (Figure 4F). Distribution maps for *F. chaus*, *P. hermaphroditus*, and *H. edwardsii* clearly showed highly suitable sites in open forests towards the south-east avoiding dense regions of the reserve, while an opposite pattern was observed in *H. vitticollis* maps, depicting its affinity towards dense canopy areas at the centre of the reserve. *V. indica* and *H. smithii*, are likely to occur in both open and moderately-close canopy areas towards the centre and south-east parts of the reserve. Suitable habitat for *F. chaus*, *P. hermaphroditus*, and *H. edwardsii* was predicted in the buffer zone (grids outside the Tiger Reserve boundary).

## Discussion

Our methodology evaluated habitat suitability models for small carnivores providing substantial improvement over traditional distribution datasets and serving as a representation of the species’ predicted areas of occupancy for practical conservation planning. The modeling results were congruent with our understanding on small carnivore natural history and their habitat preferences.

Our models depict patterns and provide an understanding of the relevant predictors (natural and anthropogenic features) that have a functional relationship with the ecology of an understudied carnivore community. Spatial distribution modeling approach identified species-specific response to environmental factors. Habitat features such as low canopy cover, less precipitation, close proximity to human habitations, are favored by *F. chaus*. The species response to important environmental predictors such as moist deciduous forest, degraded areas, and NDVI supports habitat associations known from past literature, illustrating that they frequent open savannahs, scrub jungles, moderately dense forests, agro-ecosystems, sugarcane plantations, irrigated cultivation and areas close to human habitations [42], [43]. The negative influence of NDVI on *F. chaus* was also recorded in Sariska Tiger Reserve, North-western India [10] suggesting that the species prefers open forest. The species’ morphological characteristics such as a slender body, long limbs, and a cream-coloured pelage aid the animal to camouflage well in open savannah jungles. The tufted hair on the ear tips increases sensitivity to sound which together enhances efficiency in hunting smaller prey like rodents in open forests. In Mudumalai, open forests include cropland and scrubland, which provide perfect hunting grounds for important prey species such as the Indian gerbil *Tatera indica* (Hardwicke, 1807) [44]. Throughout its distributional range, *F. chaus* is common in a wide range of habitats including dense secondary growth forests, logged areas, agricultural land and plantations (rubber tree, oil palm, sugarcane) and close to rural settlements [19], [42]. This supports the high predicted occurrence probabilities in degraded land and moist deciduous forest in our study.

*V. indica* appears to be a generalist due to its occurrence in a wide range of forests from scrubby jungles, grasslands, riverine habitats to rainforests [18]. Researchers have reported the affinity of *V. indica* to dense canopy cover and available water sources in a dry semi-arid forest of north-west India [10]. Across Southeast Asia it was recorded at low elevations and appeared to have no preference towards any forest type, although it could be more common in open habitat [45]. This supports our model results showing its affiliation with sub-tropical evergreen forest, low wetness index, and less precipitation in the reserve.

High habitat suitability in areas with less precipitation, dry deciduous and degraded forests indicate that *H. smithii* and *P. hermaphroditus* favor dry forests. The frugivorous, arboreal, and purely nocturnal habits of *P. hermaphroditus* help them adapt to a wide range of habitats including evergreen and deciduous forest (primary and secondary), plantations and near human habitation upto 2,400 m [46], [47]. In Mudumalai, *P. hermaphroditus* was not recorded in the semi-evergreen forest, but was recorded in most other forest types. Across Southeast Asia, it is found in semi-evergreen forests but appears to avoid such habitat and rainforests along the Western Ghats [46]. The species preference towards non-forest areas of Mudumalai, agrees with past occurrence records from fruit orchards, settlements, abandoned houses, agricultural lands and plantations of tea and coffee [9], [47]. Literature records reported the presence of *H. smithii* in dry forests and other forests with rocky outcrops in southern India [16]. *H. smithii* is present in varied vegetation types from arid regions in the plains of northern and western India to high altitudes (≥2,000 m) of southern India, as well as in human-dominated agricultural landscapes [50]. In Mudumalai, degraded areas and scrub forests are present at lower elevations towards the south-east region. Degraded areas are formed as a result of human activities and annual forest fires thereby providing considerably less refuge and vegetation cover for small carnivores to survive. High suitability towards degraded areas, dry deciduous forest and close proximity to villages indicates high tolerance of *H. smithii* towards human activities.

*H. vitticollis* seemed to be associated with very dense forest, sub-tropical evergreen forest, dry deciduous forest, and in areas with less precipitation in the warmest and coldest quarter. This implies that it is a forest-dwelling species, preferring moist humid and relatively cooler regions of the reserve. Frequent sightings of the animal foraging along streambeds in our study [8], [48], is indicative of the species affinity towards moist areas to fulfill dietary requirements. Along the Western Ghats, *H. vitticollis* is more common in the hills of deciduous forest (moist and dry), evergreen forests, swamps, plantations, riverine habitats, and teak plantations than distributions shown by other mongoose species [7], [49].

*H. edwardsii* appeared to prefer degraded forests in this study, which may indicate that it has a wider tolerance to disturbance than species occupying similar niches, and therefore can reach higher populations in degraded forest. The mongoose is commensal with humans as it benefits from scavenging over carrion and human refuse near human habitations and garbage dumps [51]. Throughout its range, it has been frequently recorded in dry secondary forests, thorn forests, scrublands, and cultivated fields close to water sources [51] thereby supporting its relationship with high NDVI (June) and low precipitation as observed in model outputs.

Our prediction of highly suitable habitat for *F. chaus*, *P. hermaphroditus* and *H. edwardsii* in the south-eastern parts of Mudumalai is evident given that the region is largely dominated by dry thorn and dry deciduous forests and their likely association with warm, open forests. In western regions of Mudumalai, forests become cooler, moister and denser due to the presence of semi-evergreen forests. *H. smithii* and *V. indica* were linked to dry-open and moderately-close forests, while *H. vitticollis* favored moist and dense forests.

The response curve of “distance to village” from our models must be interpreted cautiously. Although the study showed higher probability of small carnivores closest to village/tribal settlements, in reality it does not imply that these regions are suitable for the species. In most Protected Areas of India, forests close to human settlements are barren, unproductive, yet they are surrounded by natural productive habitat sufficient to fulfill ecological needs of small carnivores. In general, these carnivores may be more tolerant to human habitations than their larger cousins, or may even be forced to occupy these areas due to a high population of large carnivores [52]. Yet, it is hard to ascertain what lies behind these relationships, unless co-occurrence patterns with competing large carnivores are investigated. *V. indica* and *Herpestes spp.* are more frequent in rainforest fragments than in the relatively undisturbed, large, contiguous tract of rainforest in Kalakad–Mundanthurai Tiger Reserve [53]. Thus, small carnivores are able to persist in fragmented landscapes and degraded areas with altered community structure, but long-term persistence may require strict protection, benevolent land-use practices, and restoration. We believe this may be a pattern observed in many small mammal species [54] inhabiting continuous protected forests but may not be the case in a discontinuous fragmented landscape. Forests in the buffer area of Mudumalai are adversely impacted by cultivation, farming practices, plantations, livestock grazing, fire-wood extraction, establishment of resorts and weekend homes. Further ground validation through future surveys in the buffer zone and adjoining Reserved Forests would give a better sense of actual presence of small carnivores.

### Caveats on the use of Area under the Curve

The area under the receiver operating characteristic (ROC) curve (AUC) assesses the discriminatory capacity of species distribution models, yet, despite being the most widely used measure, several studies have severely criticized the use of AUC in species distribution models [55]–[57]. The comparison of models between species based on AUC values is flawed as there is no point in considering species with the highest AUC values to be better predicted than those with the lowest values. For example; in the present study, species with AUC value ≥0.7 does not mean that the model is “good”. A perfect model of a species niche may have a low AUC value if the species is limited by dispersal or experiences frequent local extinctions, while a model with a high AUC could be based on insignificant distinctions (e.g. an analysis of a species restricted to specialized habitat that uses a large proportion of non-specialized habitat in the background sample). Using the AUC for the evaluation of potential distribution models, or for the evaluation of species distribution models using background data instead of true absences, violates AUC theory [57]. The AUC is just one of the many metrics that can be used to evaluate the discrimination capacity of predictive models and it is only truly informative when there are true instances of absence available and the objective is the estimation of the realized distribution. Given the rate at which studies using species distribution models expand, and the importance of their potential implications in terms of conservation and management of biodiversity, an improved knowledge of the uncertainty associated with outputs of these models is important to consider in forthcoming research. The reliance on the AUC as a single measure of model performance has been seriously questioned as the AUC ignores the goodness-of-fit of the models, assumes equal costs for commission and omission errors, and is spatially independent [55], yet, it is still the most applied measure of accuracy for species distribution models and that is why we considered it for our analysis.

### Conservation Implications and Conclusions

Habitat suitability maps indicate that habitat heterogeneity driven by the east-west climatic gradient was correlated with the spatial distribution of small carnivores. Our findings bridge the knowledge gap on small carnivore ecology at small spatial extents. The application of our modeling approach could enable the identification of suitable areas where anticipation of some conservation measures is of huge importance to rare carnivores. To confirm our results and further explore the mechanisms responsible for distribution and niche patterns, field studies are needed to gather substantial data on the distribution, abundance, and ecology of small cats, civets and mongooses. Our study species appeared to be closely associated with climatic conditions and habitats to suit their ecological needs. This allows managers to preserve sufficient suitable habitat in order to sustain their populations in the near future through field management practices. Habitat modification driven by anthropogenic activities and climate change may cause range contraction of sensitive species and expansion of those tolerant to disturbances. It is feared that over the years climatic shifts might lead to rampant conversion of forests to open savannah woodland and reduction of dense evergreen forests in and around the Mudumalai landscape. *H. vitticollis* is the largest Asian mongoose with a distribution restricted to Southwest India and Sri Lanka. Widely occurring open and moderately-close forest species such as *F. chaus*, *V. indica*, *P. hermaphroditus*, *H. smithii* and *H. edwardsii* may be less vulnerable to human-caused habitat destruction when compared with closed-forest species like *H. vitticollis*. Besides, long-term occupancy of carnivores in disturbed forests or in areas with high human impact alters species interactive effects/relationships with environmental factors, as sensitivity and tolerance to habitat disturbance differs by species.

Habitat restoration in Mudumalai is recommended, keeping in mind the habitat requirements of species with specializations. Bioclimatic, topographical and anthropogenic data must be gathered in the long-term to monitor species responses to environmental variables and population trends. Although most of our study species are assigned, ‘Least Concerned’ status by IUCN, they seem to respond to disturbance and their ranges reflect climatic parameters; this necessitates the need to conduct full-fledged studies on similar species. The identification of environmental conditions associated with fine-scaled habitat variables unlikely to be captured at a landscape level, such as, predation, den-site selection, food abundance, prey distribution, and refuge habitat could be used to generate predictive spatial models by incorporating intermediate factors (such as inter/intra-species competition etc.) essential in future studies on small ranging carnivores. We hope this study will encourage researchers and conservationists to carry out similar research in other Protected Areas, fragmented forests, Reserved Forests, plantations, and urban landscapes in the country, as a basis for recording rigorous distributional data on lesser carnivores, and updating their natural history and population status.

## Supporting Information

Figure S1 **Jackknife analysis of individual predictor variables important in the development of the full model for small carnivores.** A) *F. chaus*, B) *V. indica*, C) *P. hermaphroditus*, D) *H. vitticollis*, E) *H. smithii*, and F) *H. edwardsii* in relation to the overall model quality or the “regularized training gain.” Red bars indicate the gain achieved in the jackknife results of models when including only that variable and excluding the remaining variables; green bars show how much the gain is diminished without the given predictor variable.
(TIF)

Table S1 **Pearson’s correlations between the environmental variables used in the distribution modeling for small carnivores in Mudumalai Tiger Reserve.**
(DOCX)
