# Supplementary figures and images for: Predicting the Distribution Pattern of Small Carnivores in Response to Environmental Factors in the Western Ghats
Source: PLoS One. 2013 Nov 14;8(11):e79295. doi: 10.1371/journal.pone.0079295 (PMC3828364; doi:10.1371/journal.pone.0079295)

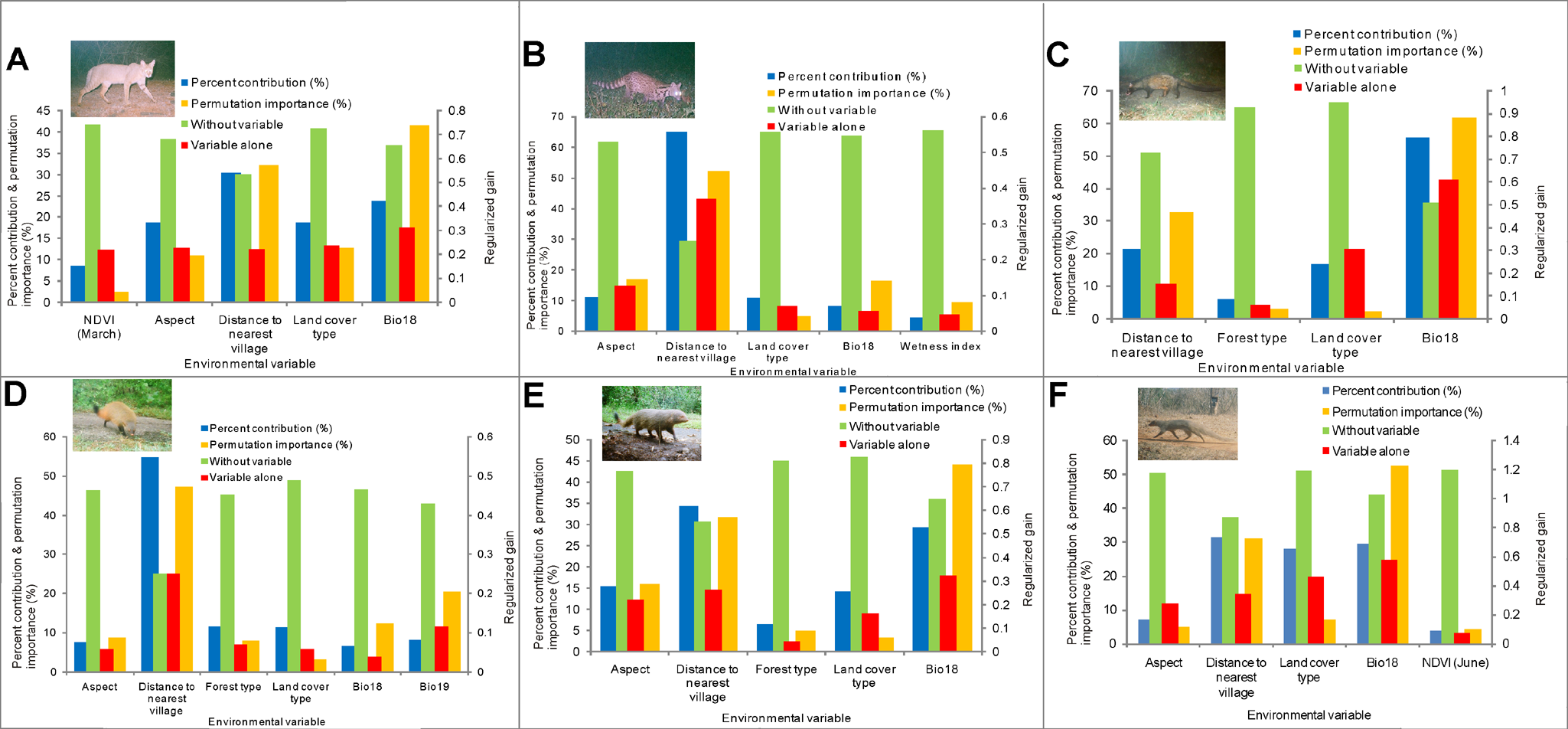

Supplement: Figure S1 — Jackknife analysis of individual predictor variables important in the development of the full model for small carnivores. A) F. chaus, B) V. indica, C) P. hermaphroditus, D) H. vitticollis, E) H. smithii, and F) H. edwardsii in relation to the overall model quality or the “regularized training gain.” Red bars indicate the gain achieved in the jackknife results of models when including only that variable and excluding the remaining variables; green bars show how much the gain is diminished without the given predictor variable. (TIF) [file pone.0079295.s001.tif]
