# Supplementary material for: Predicting the Distribution Pattern of Small Carnivores in Response to Environmental Factors in the Western Ghats
Source: PLoS One. 2013 Nov 14;8(11):e79295. doi: 10.1371/journal.pone.0079295 (PMC3828364; doi:10.1371/journal.pone.0079295)
Supplement: Table S1 — Pearson’s correlations between the environmental variables used in the distribution modeling for small carnivores in Mudumalai Tiger Reserve. (DOCX) [file pone.0079295.s002.docx]

**Table. S1** Pearson’s correlations between the environmental variables used in the distribution modeling for small carnivores in Mudumalai Tiger Reserve.

| **Variables** | **Bio3** | Bio5 | **Bio18** | **Bio19** | **NDVI**  **(March)** | **NDVI**  **(June)** | **NDVI**  **(July)** | Elevation | **Slope** | **Aspect** | **Forest type** | **Land cover category** | **WI** | **AET** | **D2W** | **D2V** |
| --- | --- | --- | --- | --- | --- | --- | --- | --- | --- | --- | --- | --- | --- | --- | --- | --- |
| **Bio3** | 1 | 0.35^**^ | -0.19^**^ | -0.03 | -0.06 | 0.07 | 0.003 | -0.38^**^ | -0.13^**^ | 0.04 | -0.11^**^ | 0.16^**^ | -0.10^**^ | -0.06 | 0.03 | -0.09^*^ |
| Bio5 | 0.35^**^ | 1 | -0.63^**^ | -0.28^**^ | -0.35^**^ | -0.14^**^ | -0.16^**^ | -0.92^**^ | -0.23^**^ | 0.15^**^ | -0.27^**^ | 0.54^**^ | -0.11^**^ | 0.06 | 0.07 | 0.01 |
| **Bio18** | -0.19^**^ | -0.63^**^ | 1 | 0.30^**^ | 0.25^**^ | 0.09^*^ | 0.10^**^ | 0.56^**^ | 0.30^**^ | -0.19^**^ | 0.11^**^ | -0.30^**^ | 0.03 | -0.25^**^ | -0.02 | 0.00 |
| **Bio19** | -0.03 | -0.28^**^ | 0.30^**^ | 1 | -0.24^**^ | 0.20^**^ | 0.17^**^ | 0.29^**^ | 0.56^**^ | 0.07 | -0.11^**^ | 0.25^**^ | 0.14^**^ | -0.26^**^ | -0.04 | 0.01 |
| **NDVI (March)** | -0.06 | -0.35^**^ | 0.25^**^ | -0.24^**^ | 1 | 0.13^**^ | 0.12^**^ | 0.23^**^ | -0.08^*^ | -0.18^**^ | 0.30^**^ | -0.61**^**^** | -0.03 | -0.04 | 0.02 | -0.04 |
| **NDVI (June)** | 0.07 | -0.14^**^ | 0.09^*^ | 0.20^**^ | 0.13^**^ | 1 | 0.40^**^ | 0.12^**^ | 0.09^*^ | 0.004 | -0.06 | -0.09^*^ | 0.03 | 0.00 | 0.01 | 0.03 |
| **NDVI (July)** | 0.003 | -0.16^**^ | 0.10^**^ | 0.17^**^ | 0.12^**^ | 0.40^**^ | 1 | 0.11^**^ | -0.01 | -0.03 | 0.02 | -0.08^*^ | 0.07^*^ | -0.09^*^ | -0.01 | -0.02 |
| Elevation | -0.38^**^ | -0.92^**^ | 0.56^**^ | 0.29^**^ | 0.23^**^ | 0.12^**^ | 0.11^**^ | 1 | 0.31^**^ | -0.06 | 0.26^**^ | -0.43^**^ | 0.13^**^ | 0.05 | -0.09^*^ | 0.01 |
| **Slope** | -0.13^**^ | -0.23^**^ | 0.30^**^ | 0.56^**^ | -0.07^*^ | 0.09^*^ | -0.01 | 0.31^**^ | 1 | -0.06 | 0.03 | 0.07 | 0.03 | -0.09^*^ | 0.00 | 0.16^**^ |
| **Aspect** | 0.04 | 0.15^**^ | -0.19^**^ | 0.07 | -0.18^**^ | 0.004 | -0.03 | -0.06 | -0.06 | 1 | -0.17^**^ | 0.21^**^ | 0.09^*^ | 0.08^*^ | 0.01 | -0.09^*^ |
| **Forest type** | -0.11^**^ | -0.27^**^ | 0.11^**^ | -0.11^**^ | 0.30^**^ | -0.06 | 0.02 | 0.26^**^ | 0.03 | -0.17^**^ | 1 | -0.29^**^ | -0.06 | 0.18^**^ | 0.01 | -0.04 |
| **Land cover category** | 0.16^**^ | 0.54^**^ | -0.30^**^ | 0.25^**^ | -0.61^**^ | -0.09^*^ | -0.08^*^ | -0.43^**^ | 0.07 | 0.21^**^ | -0.29^**^ | 1 | 0.08^*^ | 0.002 | -0.01 | 0.05 |
| **WI** | -0.10^**^ | -0.11^**^ | 0.03 | 0.14^**^ | -0.03 | 0.03 | 0.07^*^ | 0.13^**^ | 0.03 | 0.09^*^ | -0.06 | 0.08^*^ | 1 | 0.08^*^ | 0.06 | -0.03 |
| **AET** | -0.06 | 0.06 | -0.25^**^ | -0.26^**^ | -0.04 | 0.00 | -0.09^*^ | 0.05 | -0.09^*^ | 0.08^*^ | 0.18^**^ | 0.002 | 0.08^*^ | 1 | 0.01 | -0.03 |
| **D2W** | 0.03 | 0.07 | -0.02 | -0.04 | 0.02 | 0.01 | -0.01 | -0.09^*^ | 0.00 | 0.01 | 0.01 | -0.01 | 0.06 | 0.01 | 1 | 0.02 |
| **D2V** | -0.01^*^ | 0.01 | 0.00 | 0.01 | -0.04 | 0.03 | -0.02 | 0.01 | 0.16^**^ | -0.09^*^ | -0.04 | 0.05 | -0.03 | -0.03 | 0.02 | 1 |

Isothermality (Bio3), Max Temperature of the Warmest Month (Bio5), Precipitation of the warmest quarter (Bio18), Precipitation of the coldest quarter (Bio19), Normalized Difference Vegetation Index for March, June, July (NDVI), Topography Wetness Index (WI), Actual evapo-transpiration (AET), Distance to the nearest water source (D2W), and Distance to the nearest village/tribal settlement (D2V).

**. Correlation is significant at the 0.01 level (2-tailed).

Correlated variables, elevation and Bio5 were removed from ecological niche models for each species.
